# Preoperative psychological assessment for bariatric surgery in the Brazilian public health system

**DOI:** 10.1192/j.eurpsy.2025.1414

**Published:** 2025-08-26

**Authors:** A. A. Lemos, C. M. Trentini, E. R. Marcon, J. U. Castan

**Affiliations:** 1 Psychology, UFRGS; 2 Physical Education; 3 Psychology, HCPA, Porto Alegre, Brazil

## Abstract

**Introduction:**

The Preoperative Psychological Assessment of Bariatric Surgery and Metabolic (PP-BSM) aims to detect psychopathologies before the procedure, facilitating interventions and lifestyle changes.

**Objectives:**

Describing and analyzing the psychological aspects and tools used for pre-bariatric surgery psychological evaluation in the Brazilian Public Health System.

**Methods:**

Descriptive cross-sectional study. The convenience sample was composed by psychologists of the bariatric surgery and metabolic teams in the public health system in a region in the South of Brazil. Data were collected through an electronic questionnaire administered via telephone survey, focusing on PP-BSM.

**Results:**

There was consensus regarding the areas to be assessed: cognition, emotion, social support, and psychiatric comorbidities. Variation was found in the protocols and instruments used for PP-BSM, with each hospital using its own protocol. Semi-structured clinical interviews, along with self-reported scales and questionnaires, were used by most psychologists.

**Conclusions:**

Although the psychological aspects assessed are aligned with current Brazilian guidelines, there is a lack of either standardized protocols among services or specific instruments for bariatric surgery candidates. Providing adequate tools and training professionals in their use may contribute to increasing safety and long-term positive surgical outcomes.

**Disclosure of Interest:**

None Declared

